# Comparison of the Probiotic Potential between *Lactiplantibacillus plantarum* Isolated from Kimchi and Standard Probiotic Strains Isolated from Different Sources

**DOI:** 10.3390/foods10092125

**Published:** 2021-09-08

**Authors:** Chang-Hee Jeong, Hyejin Sohn, Hyelyeon Hwang, Ho-Jae Lee, Tae-Woon Kim, Dong-Sub Kim, Chun-Sung Kim, Sung-Gu Han, Sung-Wook Hong

**Affiliations:** 1 Microbiology and Functionality Research Group, World Institute of Kimchi, Gwangju 61755, Korea; jeongch@wikim.re.kr (C.-H.J.); hyelyeon@wikim.re.kr (H.H.); dlghwo321@wikim.re.kr (H.-J.L.); korkimchiman@wikim.re.kr (T.-W.K.); 2 Department of Food Science and Biotechnology of Animal Resources, Konkuk University, Seoul 05029, Korea; sonhjin123@konkuk.ac.kr (H.S.); hansg@konkuk.ac.kr (S.-G.H.); 3 Research Institute, Korea Prime Pharm. Co., Ltd., Gwangju 61473, Korea; ds.kim@koreaprime.co.kr; 4 Department of Oral Biochemistry, College of Dentistry, Chosun University, Gwangju 61452, Korea; cskim2@chosun.ac.kr

**Keywords:** probiotic properties, *Lactiplantibacillus plantarum*, kimchi, anti-inflammatory activity

## Abstract

In the present study, the properties of the *Lactiplantibacillus* (*Lpb.*) *plantarum* WiKim0112 isolated from kimchi were evaluated by comparing its probiotic properties to those of *Lpb. plantarum* WCFS1 and KACC 11451 isolated from different sources. In both pH 2 and 3, media containing pepsin, Wikim0112, and WCFS1 showed higher cell viability than KACC11451. Viability of all *Lpb*. *plantarum* strains in a medium containing pancreatin and bile salt oxgall was significantly decreased compared to the control. WCFS1 showed the highest thermotolerance, followed by Wikim0112 and KACC11451. Wikim0112 showed a similar level of antibacterial activity to WCFS1 and exhibited an overall higher antibacterial activity than KACC11451 against six pathogens. All *Lpb. plantatum* strains showed high antioxidant activities in SOD, DPPH, and ABTS assays, especially Wikim0112 and WCFS1 exhibited a higher antioxidant activity than KACC11451. All *Lpb*. *plantarum* strains showed approximately 60–62% adhesion rates to Caco-2 cells. Moreover, in LPS-stimulated Caco-2 cells, all *Lpb*. *plantarum* strains significantly decreased the mRNA expression of pro-inflammatory cytokines (i.e., IL-1β, IL-6, and TNF-α); Wikim0112 significantly increased the mRNA expression of IL-4 and IFN-γ. Wikim0112 was resistant to streptomycin and vancomycin, whereas WCFS1 and KACC11451 were resistant to four (clindamycin, ciprofloxacin, tetracycline, and vancomycin) and three (ciprofloxacin, tetracycline, and vancomycin) antibiotics, respectively. These results, taken together, indicated that compared to *Lpb*. *plantarum* strains isolated from different sources, Wikim0112 showed desirable probiotic properties, suggesting its potential applications in the food and pharmaceutical industries.

## 1. Introduction

Kimchi is a traditional Korean fermented vegetable food that is prepared by fermenting salted vegetables and various ingredients, including red pepper powder, garlic, and ginger using lactic acid bacteria (LAB). Among the LAB used in kimchi production, members of the genera *Leuconostoc*, *Weissella*, and *Lactobacillus* are known to play a critical role in kimchi fermentation [1]. Kimchi-derived LAB have been reported to have beneficial effects on human health. According to a previous study, *Lactobacillus acidophilus* KFRI342 isolated from kimchi exhibited probiotic properties suppressing human colon cancer cell growth [2]. *Levilactobacillus brevis* KU15147 isolated from kimchi showed higher antioxidant activity than that of *Lactobacillus* *rhamnosus* GG used as a reference strain; the KU15147 strain also showed immune-enhancing effects via nitric oxide (NO) production and cytokine induction (i.e., inducible NO synthase, and tumor necrosis factor-α (TNF- α)) [3].

*Lactiplantibacillus* (*Lpb.*) *plantarum*, which is abundant in kimchi, represents an ideal candidate for developing probiotics owing to remarkable adhesion and adaptation capabilities in the gastrointestinal (GI) tract and beneficial effects such as antimicrobial, anti-inflammatory, and antioxidant activities [4]. *Lpb. plantarum* strains have been widely used in the food industry as starter cultures in the fermentation of foods and beverages as they provide organoleptic properties, texture, and flavor [5]. Thus, they are isolated from various sources such as fermented vegetables [6,7,8], milk products [9,10], and meat [11,12], and their functional characteristics, including probiotic potential, have been evaluated.

However, single species have been found to show differences in characteristics based on the isolation source [13,14]. Therefore, in this study *Lpb. plantarum* WiKim0112 isolated from Korean radish water kimchi was evaluated by comparing its probiotic properties to two *Lpb. plantarum* strains isolated from different sources.

## 2. Materials and Methods

### 2.1. Bacterial Strains and Growth Conditions

Two *Lpb. plantarum* strains showing desirable probiotic features included *Lpb. plantarum* WCFS1 ATCC BAA-793 isolated from human saliva and *Lpb. plantarum* KACC11451 isolated from pickles that were obtained from the American Type Culture Collection (ATCC) and Korean Agricultural Culture Collection (KACC), respectively. *Lpb.* *plantarum* 8 species (WiKim0112, DK4, YB5, JC8, YP2, PC2, TS3, LB10), *Lacticaseibacillus casei* 5 species (KCKM0991, DO1, T18, PC2, LB2), *Lacticaseibacillus paracasei* 5 species (KCKM1011, L01, LB17, FS31, TS6), and *Limosilactobacillus fermentum* 2 species (KCKM0729, PC5) isolated from kimchi and obtained from the Korean Collection for Kimchi Microorganisms (KCMC) were cultured anaerobically in de Man, Rogosa, and Sharpe (MRS) broth for 48 h at 30 °C. Six bacterial pathogens obtained from ATCC and the Korean Culture Center of Microorganisms (KCCM) included: *Escherichia coli* O157:H7 (ATCC 35150), *Listeria monocytogenes* (ATCC 15313), *Salmonella choleraesuis* (KCCM40763), *Shigella boydii* (KCCM41649), *Staphylococcus aureus* (ATCC25923), and *Yersinia enterocolitica* (KCCM41657). All strains were cultured in tryptic soy broth (TSB; Difco, Sparks, MD, USA) for 24 h at 30 °C and stored in a TSB medium containing 20% glycerol at −70 °C.

### 2.2. Cell Culture

The monocyte/macrophage cell line RAW264.7 and the human colorectal adenocarcinoma cell line Caco-2 were obtained from the Korean Cell Line Bank (Jongno, Seoul, Korea). The cells were maintained in Dulbecco’s modified Eagle’s medium (DMEM; Welgene Inc., Gyeongsan, Korea) containing 10% fetal bovine serum (Welgene Inc., Gyeongsan, Korea) and antibiotics at 37 °C in a humidified atmosphere containing 5% CO_2_. The medium was replaced with fresh medium every 2–3 d. The cells were digested using 0.05% trypsin/0.53 mM ethylenediaminetetraacetic acid (Welgene Inc., Gyeongsan, Korea) when they reached 80% confluency in T-25 flasks. The cells were then seeded in 6-well plates.

### 2.3. Selection of Bacteria Showing Anti-Inflammatory Activity

RAW264.7 cells (1 × 10^5^ cells/mL) were seeded in 6-well plates, incubated overnight, and then exposed to lipopolysaccharide (LPS; 100 ng/mL) for 24 h. Culture supernatants of isolates were collected via centrifugation at 3800× *g* (4 °C) for 15 min. The levels of TNF-α, interleukin (IL)-6, and IL-1β secreted into the culture supernatants were determined in duplicate using respective Quantikine enzyme-linked immunosorbent assay (ELISA) kits (R&D Systems Inc., Minneapolis, MN, USA) according to the manufacturer’s instructions. The concentration of NO in the cell culture supernatant was determined using the Griess reagent (Sigma-Aldrich, St. Louis, MO, USA). The culture supernatant (50 μL) was mixed with an equal volume of Griess reagent and incubated for 15 min at room temperature (25–28 °C). Absorbance (540 nm) was measured using an ELISA microplate reader Infinite 200 pro (Tecan Austria GmbH, Grödig, Austria).

### 2.4. Identification of the Strains

Chromosomal DNA was extracted from bacteria using a commercial QIAamp DNA Mini Kit (Qiagen, Hilden, Germany) according to the manufacturer’s instructions. The conserved region of the 16S rRNA gene was amplified with the universal primer pairs (FP: (27F) 5′-AGAGTTTGATCCTGGCTCAG-3′ and RP: (1492R) 5′-GGTTACCTTGTTACGACTT-3′) using an Applied biosystems thermal cycler (Thermo Fisher Scientific, Pittsburgh, PA, USA). Polymerase chain reaction (PCR) amplification was performed in 20 μL of final reaction volume containing 18 μL of PCR master mix and 2 μL of DNA sample. The PCR reaction conditions were as follows: initial denaturation for 4 min at 94 °C, followed by 35 cycles of denaturation for 1 min at 94 °C, primer annealing for 1 min at 57 °C, extension for 1 min at 72 °C, and a final elongation for 10 min at 72 °C. The amplified PCR products were sequenced using ABI 3730XL Genetic Analyzer (Applied Biosystems, Waltham, MA, USA). The assembled 16S rRNA gene sequence was identified using the EZBioCloud database (www.ezbiocloud.net/eztaxon, accessed on 10 May 2021) and compared to closely related strains.

### 2.5. Tolerance of GI Environment and Heat

Tolerance of the *Lpb. plantarum* strains to the GI environment was determined as previously described by Ranadheera et al. (2012) [15], with certain modifications. MRS broth was added with or without 0.3% (*w*/*v*) pepsin (Sigma Aldrich, St. Louis, MO, USA), and the pH was adjusted to 2, 3, or 6.5 (control) using 1 N HCl to evaluate gastric juice tolerance. MRS broth was supplemented with or without (control) 0.1% (*w*/*v*) pancreatin (Sigma Aldrich, St. Louis, MO, USA) and 0.3% (*w*/*v*) bile salts (Sigma Aldrich, St. Louis, MO, USA), and the pH was adjusted to 7.5 using1N NaOH to evaluate small intestinal juice tolerance. Overnight cultured *Lpb. plantarum* strains were inoculated into simulated gastric and small intestinal juice solutions at a density of 1 × 10^7^ colony forming units (CFU)/mL and then incubated at 37 °C for 4 h and 24 h, respectively. To assess the thermotolerance of the *Lpb. plantarum* strains, the inoculated MRS broths (1 × 10^7^ CFU/mL) were incubated at 50 °C for 1 h. After incubation, the MRS broths containing bacteria were serially diluted and spread onto MRS agar plates, followed by incubation at 30 °C for 48 h. The number of colonies formed on the MRS agar plates was counted using the plate counting method. For all tolerance tests, the bacterial survival was calculated using the following formula:

Survival (%) = (treatment group CFU/mL)/(control group CFU/mL) × 100

### 2.6. Antibacterial Activity

The antibacterial activity was evaluated using the well diffusion method. Soft tryptic soy agar (TSA) was seeded with pathogenic bacteria (1 × 10^5^ CFU/mL). Wells of 8 mm in diameter were created on the TSA agar plates using a sterilized cork borer. Culture supernatants were prepared via centrifugation (3800× *g*, 4 °C, 15 min) and used as an antagonistic substance. Culture supernatants 100 μL were poured into the wells and subsequently incubated at 30 °C for 24 h. The diameter of the zone of growth inhibition was measured in millimeters to evaluate the antagonistic effects. MRS broth was used as the negative control.

### 2.7. Antioxidant Activity

The antioxidative activity of the *Lpb. plantarum* strains was estimated by superoxide dismutase (SOD), 2,2-diphenyl-1-picryl-hydrazyl (DPPH), and 2,2′-azino-bis(3-ethylbenzothiazoline-6-sulphonic acid) (ABTS) assays. The *Lpb. plantarum* strains were cultured in a minimal medium (1% (*w*/*v*) glucose containing 0.5% (*w*/*v*) yeast extract) at 30 °C for 48 h. After centrifugation (8000× *g*, 15 min), the supernatant was used as a sample to measure the antioxidant activity.

The SOD assay was performed according to the manual of the Amplite Colorimetric SOD Assay Kit (AAT Bioquest, Sunnyvale, CA, USA). In the assay, xanthine is converted to hydrogen peroxide (H_2_O_2_) radical ions, uric acid, and H_2_O_2_ by xanthine oxidase. The degree of oxidation of xanthine, which catalyzes the conversion to H_2_O_2_, was measured and interpreted as SOD-like activity. The sample (50 μL) was mixed with a reagent (50 μL) containing xanthine and xanthine oxidase and reacted at room temperature for 30 min. Subsequently, the absorbance was measured at 450 nm using an ELISA microplate reader Infinite 200 pro (Tecan Austria GmbH, Grödig, Austria), and SOD-like activity was expressed as follows:

SOD-like activity (%) = [1 − (OD_sample_/OD_blank_) × 100]

The DPPH assay was conducted as described previously with certain modifications [16]. Briefly, 100 μL of *Lpb. plantarum* supernatant was mixed with 100 μL of the DPPH reagent in a 96-well plate. After incubation in the dark at room temperature for 30 min, the absorbance was measured at 517 nm using an ELISA microplate reader Infinite 200 pro (Tecan Austria GmbH, Grödig, Austria). As a positive control, 0.5% L-ascorbic acid was added in the same amount as that of the sample.

The ABTS assay was performed as previously described with certain modifications [17]. ABTS was dissolved in 50% ethanol at a concentration of 70.0 mM and mixed with 24.5 mM potassium persulfate; the reaction occurred in the dark for 12 h. After the reaction, the solution was diluted to obtain an absorbance value of 0.7–0.8 ± 0.02 at 734 nm. The supernatant of the *Lpb. plantarum* strains (100 μL) was mixed with 100 μL of ABTS^+^ solution in a 96-well plate. After incubation in the dark at room temperature for 15 min, the absorbance was measured at 734 nm using an ELISA microplate reader Infinite 200 pro (Tecan Austria GmbH, Grödig, Austria). As a positive control, 0.5% L-ascorbic acid was added in the same amount as that of the sample. DPPH and ABTS radical scavenging activities (%) were calculated as follows:

Radical scavenging activity (%) = [1 − (OD_sample_/OD_control_) × 100]

### 2.8. Adhesion Ability to Intestinal Epithelial Cells

The adhesion ability of the *Lpb. plantarum* strains to intestinal epithelial cells was evaluated using Caco-2 cells as described in a previous study [18]. Briefly, overnight cultures of the *Lpb. plantarum* strains were centrifuged at 4 °C (6000× *g*, 10 min). The pellet was washed with phosphate-buffered saline (PBS) and resuspended in antibiotic-free DMEM. A monolayer of Caco-2 cells (80–90% confluency) was treated with bacterial suspensions (1 × 10^7^ CFU/mL), followed by incubation at 37 °C for 2 h. The cells were then rinsed with PBS thrice to remove non-adherent bacteria. Next, Caco-2 cells were detached using trypsin (Welgene Inc., Gyeongsan, Korea) and lysed with 0.1% Triton X-100 (Sigma Aldrich, St. Louis, MO, USA). The serially diluted cell lysates were spread onto MRS agar plates and incubated at 37 °C for 24 h. After incubation, the number of colonies formed on MRS agar plates was counted by the plate counting method.

### 2.9. Anti-Inflammatory Activity

To evaluate the effects of *Lpb. plantarum* strains on the gene expression of inflammatory cytokines [TNF-α, IL-6, IL-1β, IL-4, IL-10, and interferon-gamma (IFN-γ)], quantitative real-time PCR was performed. Caco-2 cells were treated with 1 × 10^7^ CFU/mL of *Lpb. plantarum* strains in antibiotic-free DMEM for 6 h. The medium containing bacteria was removed from the well plates, and the cells were exposed to LPS (1 μL/mL) for 2 h to induce an inflammatory response. Total RNA was extracted from the cells using the TRIzol reagent (Ambion, Austin, TX, USA). Reverse transcription was then performed using the TOPscript RT DryMIX kit (Enzynomics, Daejeon, Korea) according to the manufacturer’s protocol. The mRNA expression levels were measured by real-time PCR using the Roche LightCycler^®^ 96 System (Roche, Basel, Switzerland) and a real-time PCR mix (SolGent, Daejeon, Korea). The thermal cycling conditions for PCR were as follows: initial denaturation at 95 °C for 15 min (enzyme activation), followed by 40 cycles at 95 °C for 10 s, 60 °C for 10 s, and 72 °C for 10 s. The mRNA expression was relatively quantified using the ΔΔCq method; glyceraldehyde 3-phosphate dehydrogenase (GAPDH) was used as the housekeeping gene. The primers designed are shown in Table 1.

### 2.10. Antibiotic Susceptibility

Antibiotic susceptibility of the *Lpb. plantarum* strains was investigated as described previously [19]. A total of 13 antibiotics were used to evaluate antibiotic resistance: ampicillin (AMP; 10 μg), chloramphenicol (C; 30 μg), clindamycin (CD; 10 μg), ciprofloxacin (CIP; 5 μg), gentamicin (CN; 10 μg), doxycycline (DXT; 30 μg), erythromycin (E; 15 μg), kanamycin (K; 30 μg), penicillin G (P; 10 IU), streptomycin (S; 10 μg), trimethoprim-sulfamethoxazole (SXT; 25 μg), tetracycline (TE; 30 μg), and vancomycin (VA; 30 μg) (KisanBio, Seoul, Korea). Each *Lpb. plantarum* strain (1 × 10^7^ CFU/mL; 100 μL) was spread onto MRS agar plates, and antibiotic discs were placed on the plates. After incubation at 37 °C for 24 h, the diameter of the inhibition zone was measured. The results were presented according to the Clinical and Laboratory Standards Institute 2012 standards [20].

### 2.11. Statistical Analysis

Data are presented as the mean ± standard deviation (SD) or standard error of the mean (SEM) (n = 3 or 4). Statistical significance was determined using the independent two-sample t-test and Tukey’s post hoc test. The tests were performed using the Predictive Analytics Software statistics for Windows (version 19.0; SPSS, Chicago, IL, USA). A value of *p* < 0.05 was considered significant.

## 3. Results and Discussion

### 3.1. Selection of Bacteria with Anti-Inflammatory Activity

Twenty kimchi-fermenting microorganisms, including *Lpb. plantarum*, *Lacticaseibacillus casei*, *Lacticaseibacillus paracasei*, and *Limosilactobacillus fermentum* were screened for potential probiotic strains. Among them, strain Wikim0112 decreased the levels of pro-inflammatory cytokines including TNF-α, IL-6, and IL-1β, and NO in LPS-stimulated RAW264.7 cells (data not shown).

### 3.2. Identification of WiKim0112

**Figure 1:**
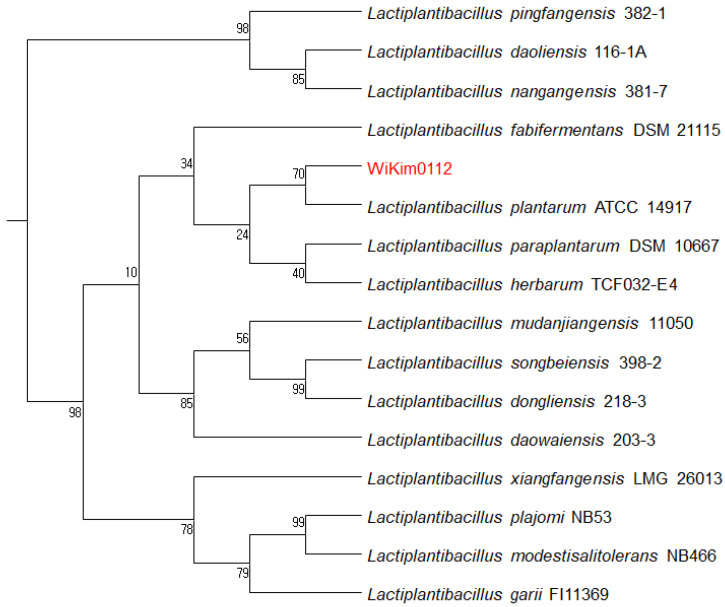
Phylogenetic tree constructed using neighbor-joining based on 16S rRNA gene sequences of WiKim0112. Bootstrap percentages for 1000 re-sampling are given.

A phylogenetic tree was constructed based on the 16S rRNA sequences to identify strain Wikim0112 and investigate the evolutionary relationships. The 16S rRNA sequence of WiKim0112 was compared to similar sequences using the EZBioCloud database. The sequence showed high similarity to *Lpb.* *plantarum* (16S rRNA sequence similarity, >99%). According to the phylogenetic tree, the isolated WiKim0112 was closely related to *Lpb.* *plantarum* ATCC 14917, with bootstrap values of 70% (Figure 1).

### 3.3. Viability of Lpb. plantarum Strains to GI Environment and Heat Treatment

**Figure 2:**
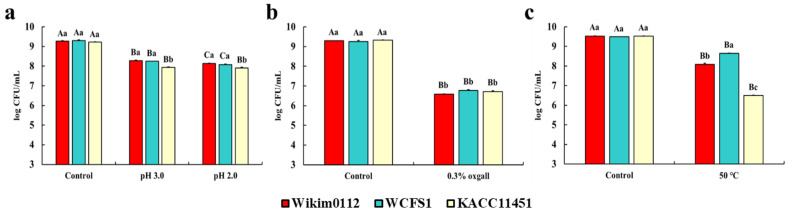
Gastrointestinal environment and heat tolerance of *Lpb. plantarum* Wikim0112, WCFS1, and KACC11451. All *Lpb.* *plantarum* strains were exposed (**a**) without (control) or with 0.3% (*w*/*v*) pepsin at pH 6.5 (control), 3.0, or 2.0 for 4 h, (**b**) without (control) or with 0.1% (*w*/*v*) pancreatin and 0.3% (*w*/*v*) bile salt (oxgall) for 24 h, and (**c**) 30 (control) or 50 °C for 1 h. The values represent the mean ± SEM (n = 3). Different uppercase letters represent significant difference between the same strains (*p* < 0.5), while different lowercase letters represent significant difference between the same treatment groups (*p* < 0.5).

Tolerance to the GI environment, including acidic juice containing pepsin, bile, and pancreatin, is a valuable property of probiotics that is associated with beneficial effects on host health [21]. In particular, acid tolerance is important in bacteria not only for enduring gastric stress but also for extending the duration of survival of the bacteria in acidic foods such as yogurt [22]. Since the pH of gastric juice after food ingestion is known to be approximately 3, we used pH 2 and 3 media containing pepsin in our study to mimic gastric juice [23]. In addition, considering the general bile salt concentration of the human GI tract (0.03–0.3%), 0.3% bile salt oxgall and pancreatin were incorporated in the culture media [24]. The tolerance of *Lpb. plantarum* Wikim0112, *Lpb. plantarum* WCFS1, and *Lpb. plantarum* KACC11451 to the GI environment is presented in Figure 2. All *Lpb. plantarum* strains showed relatively high cell viabilities of 8.28 log CFU/mL for Wikim0112, 8.25 log CFU/mL for WCFS1, and 7.93 log CFU/mL for KACC11451 in pH 3 medium containing 0.3% pepsin, compared to the control (9.22–9.29 log CFU/mL, pH 6.5, without pepsin) (Figure 2a). In particular, Wikim0112 and WCFS1 showed significantly higher viability than that of KACC11451 (Figure 2a). Moreover, in a pH 2 medium containing pepsin, Wikim0112 (8.14 log CFU/mL), WCFS1 (8.01 log CFU/mL), and KACC11451 (7.91 log CFU/mL) showed relatively high cell viability, and KACC11451 showed significantly lower viability than that of the other two strains (Figure 2a). This difference in acid resistance in the same species may be attributed to the relative amount of H^+^-ATPase present in the cell membranes [25]. H^+^-ATPase controls proton permeability, allowing cells to withstand large differences in pH between the bacterial cytoplasm and the medium [26]. The bile tolerance of *Lpb. plantarum* strains were investigated using a medium containing 0.1% pancreatin and 0.3% bile salt oxgall. The cell viability of all *Lpb. plantarum* strains was significantly decreased to 6.58–6.77 log CFU/mL, compared to 9.26–9.32 log CFU/mL of the control (without pancreatin and bile salt) (Figure 2b). However, there were no significant differences between the strains.

The thermotolerance of probiotics may facilitate their survival during thermal food processing. Therefore, we analyzed the survival of the strains at 50 °C to evaluate their thermotolerance. We found that WCFS1 (8.65 log CFU/mL) showed the highest thermotolerance, followed by Wikim0112 (8.14 log CFU/mL) and KACC (6.45 log CFU/mL) compared to the initial cell density (9.50–9.53 log CFU/mL) (Figure 2c). This result may be attributed to the fact that WCFS1 was isolated from human saliva, the temperature of which is relatively higher than that of the isolation sources of the other two strains. Similarly, a previous study reported that LAB isolated from cooked meat products showed thermotolerance [27].

### 3.4. Antibacterial Activity of Lpb. plantarum Strains

*Lpb. plantarum* strains are known to produce a wide range of antibacterial compounds, including bacteriocins, organic acids, ethanol, diacetyl, and H_2_O_2_ [28,29,30]. Furthermore, antibacterial substances produced by LAB are generally recognized as non-toxic and non-irritant components because they are naturally degraded in the body or the environment [31,32]. Therefore, antibacterial activity is an important property of probiotics. The antibacterial activity of cell-free culture supernatants of *Lpb. plantarum* strains against six strains of foodborne bacterial pathogens are shown in Table 2. Wikim0112 showed a similar level of antibacterial activity to that of the reference strain WCFS1 and exhibited an overall higher antibacterial activity than that of KACC11451 against six pathogens. Our results suggest that Wikim0112 may show an antibacterial activity that is not inferior to that of the two commercial *Lpb. plantarum* strains, particularly against bacterial pathogens associated with foodborne diseases.

### 3.5. Antioxidant Activity of Lpb. plantarum Strains

**Figure 3:**
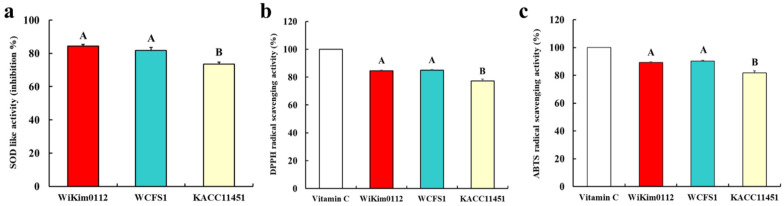
Antioxidative activities of *Lpb. plantarum* Wikim0112, WCFS1, and KACC11451. (**a**) Superoxide dismutase (SOD)-like activity, (**b**) 2,2-diphenyl-1-picryl-hydrazyl (DPPH), and (**c**) 2-azino-bis(3-Ethylbenzothiazoline-6-Sulfonic Acid) (ABTS) scavenging activities were estimated. As a positive control, 0.5% L-ascorbic acid was used with the same amount as the sample. The values represent the mean ± SD (n = 3). Different letters represent significant difference (*p* < 0.5).

A previous study showed that the antioxidant effects of probiotics might be mediated via the chelation of metal ions, expression of anti-oxidases, generation of antioxidant metabolites, modulation of anti-oxidase activity in the host, and regulation of the gut microbiota [33]. In the present study, we determined the antioxidant activity of the *Lpb. plantarum* strains via SOD, DPPH, and ABTS assays. All *Lpb*. *plantatum* strains showed more than 70% of SOD-like activity, especially Wikim0112 (84.3%) and WCFS1 (81.7%) had higher SOD-like activity than KACC11451(73.5%) (Figure 3a). Moreover, in DPPH and ABTS assay, Wikim0112 and WCFS1 showed significantly higher radical scavenging activities than KACC11451 (Figure 3b,c). A previous study reported that *Lpb. plantarum* C88 isolated from traditional Chinese fermented foods demonstrated DPPH scavenging activity of 44.3% [34]. In particular, heat-killed *Lpb. plantarum* Ln1 isolated from kimchi showed a high ABTS radical scavenging activity of 70.2% [35]. Our results indicate that Wikim0112 had similar or stronger antioxidant activity than standard probiotic strains.

### 3.6. Adhesion Ability of Lpb. plantarum Strains to Intestinal Epithelial Cells

**Figure 4:**
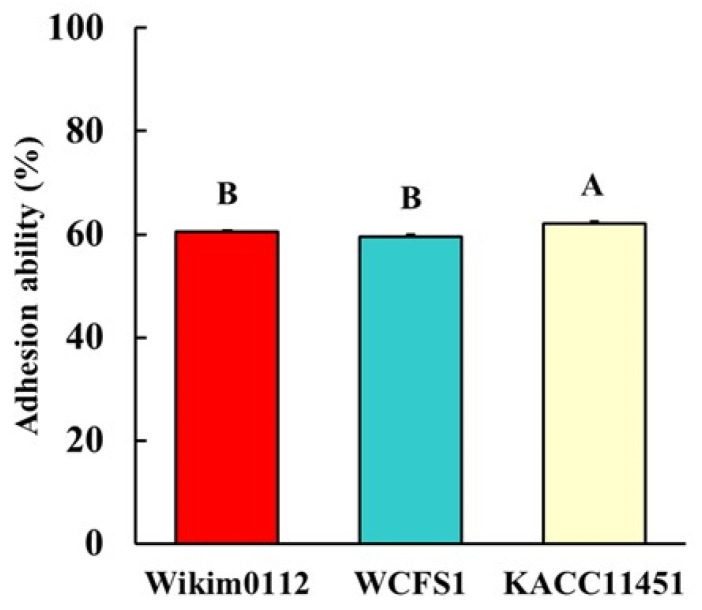
The adhesion ability of *Lpb. plantarum* Wikim0112, WCFS1, and KACC11451 in Caco-2 cells. The cells were exposed to *Lpb. plantarum* strain at 37 °C for 2 h. The adhesion rates were calculated by counting the number of bacteria before and after incubation. The values represent the mean ± SEM (n = 4). Different letters represent significant difference (*p* < 0.5).

Probiotics may provide beneficial health effects by inhibiting the colonization of pathogens and enhancing mucosal healing during adhesion to the intestinal epithelium [36]. Therefore, the ability of probiotics to adhere to the intestinal epithelium has been evaluated. In general, the Caco-2 cell line has been commonly used in assays to estimate the adhesion ability of potential probiotic microorganisms due to its functional differentiation in vitro and morphological characteristics similar to those of human enterocytes [37,38]. The capability of the selected *Lpb. plantarum* strains to adhere to the intestinal epithelium was estimated by incubating the cultures with Caco-2 cells for 2 h. All *Lpb. plantarum* strains showed adhesion rates of approximately 60–62% (Figure 4). In particular, KACC11451 showed a higher adhesion ability than that of Wikim0112 and WCFS1 (Figure 4). The adhesion ability of probiotics may be affected by several factors, such as non-specific binding, including electrostatic and hydrophobic interactions, and specific binding to molecules, such as the mannose-specific binding of strain WCFS1 [39,40]. Therefore, isolates of the same microbial species may differ in their capability to adhere to the intestinal epithelium.

### 3.7. Anti-Inflammatory Activity of Lpb. plantarum Strains in Intestinal Epithelial Cells

**Figure 5:**
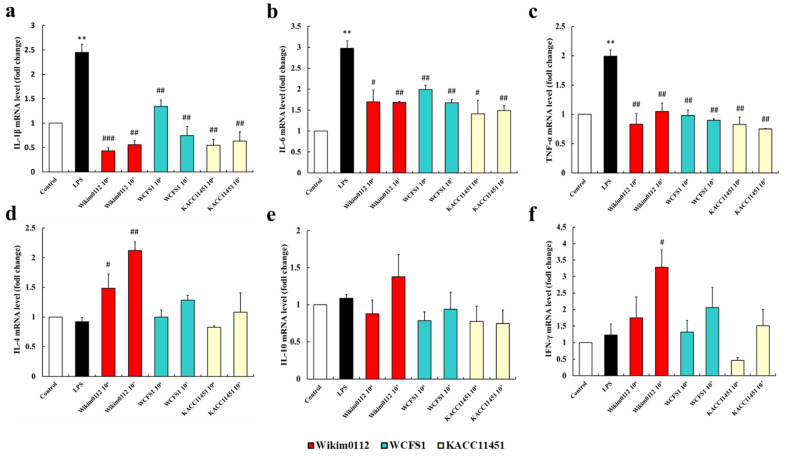
Effects of *Lpb. plantarum* Wikim0112, WCFS1, and KACC1451 on the mRNA expression levels of (**a**–**c**) pro-inflammatory cytokines (IL-1β, IL-6, and TNF-α) and (**d**–**f**) anti-inflammatory cytokines (IL-4, IL-10, and IFN-γ) in Caco-2 cells. The cells were pretreated with each *Lpb. plantarum* strain (10^6^ and 10^7^ CFU/mL) for 6 h followed by treatment with LPS (1 μL/mL) for 2 h. The values represent the mean ± SEM (n = 3); ** *p* < 0.01 indicates a significant difference vs. the control and ^#^ *p* < 0.05, ^##^ *p* < 0.01, ^###^ *p* < 0.001 indicates a significant difference vs. the LPS-treated group.

Probiotics adhered to the intestinal epithelium may exhibit immunomodulatory activity by regulating pro- and anti-inflammatory cytokine production and inducing peripheral immunoglobulin production and immunoglobulin A secretion [41]. We found that all *Lpb. plantarum* strains significantly decreased the mRNA expression levels of pro-inflammatory cytokines (IL-1β, IL-6, and TNF-α) in LPS-stimulated Caco-2 cells (Figure 5a–c). The WCFS1 and KACC11451 strains did not affect the mRNA expression of anti-inflammatory cytokines (Figure 5d–f), whereas Wikim0112 significantly increased the mRNA expression of IL-4 and IFN-γ in LPS-stimulated Caco-2 cells (Figure 5d,f). Another study similarly reported anti-inflammatory activity in LAB isolated from kimchi. Park et al. (2017) found that administration of Lactobacillus paracasei LS2 isolated from kimchi significantly decreased the mRNA levels of pro-inflammatory cytokines (TNF-α, IL-1β, and IL-6) in dextran sulfate sodium-induced colitis mice [42]. Our results indicated that the Wikim0112 strain isolated from kimchi showed higher anti-inflammatory activity than that of the other two strains.

### 3.8. Antibiotic Susceptibility of Lpb. plantarum Strains

Antibiotic resistance of bacteria is largely classified into intrinsic and acquired resistance (for example, by spontaneous mutations in the bacterial genome or acquisition of additional genes) [43,44]. According to previous reports, the intrinsic resistance of bacteria was not transferred horizontally and did not present a risk in non-pathogenic bacteria, whereas acquired resistance can be horizontally transferred between bacteria [43,44]. Thus, it is essential to evaluate the antibiotic resistance of LAB. Antibiotic susceptibility of the *Lpb. plantarum* strains was determined using 13 antibiotics (AMP, C, CD, CIP, CN, DXT, E, K, P, S, SXT, TE, and VA). Wikim0112 was resistant to two antibiotics (S and VA) (Table 3). WCFS1 and KACC11451 were resistant to four (CD, CIP, TE, and VA) and three (CIP, TE, and VA) antibiotics, respectively (Table 3). The *Lpb. plantarum* strains are generally susceptible to β-lactam antibiotics, which inhibit the cell wall synthesis, such as ampicillin and penicillin, except for cephalosporins and oxacillin [44]. However, they are usually resistant to glycopeptides, such as VA [44]. Similarly, we found that Wikim0112 showed resistance to glycopeptides (VA) and aminoglycoside antibiotics (S), which are known to be intrinsic to Lactiplantibacilli [45,46]. These results indicated that Wikim0112 might not present a risk to the host compared to the other two standard probiotic strains regarding antibiotic resistance.

## 4. Conclusions

Compared to the probiotic *Lpb*. *plantarum* strains isolated from human saliva and pickle, kimchi-derived *Lpb*. *plantarum* Wikim0112 exhibited desirable probiotic properties, such as heat tolerance, resistance to the GI environment, and adhesion to the intestinal epithelium, along with antibacterial, antioxidant, and anti-inflammatory activities. Although further studies on the safety of *Lpb. plantarum* Wikim0112 are warranted, Wikim0112 represented an ideal candidate for probiotic development in the food and pharmaceutical industries.

## Figures and Tables

**Table 1:** Primers used for real-time PCR.

Genes	Primer Sequences (5′–3′)
TNF-α ^a^	(F) AAG CCC TGG TAT GAG CCC ATC TAT(R) AGG GCA ATG ATC CCA AAG TAG ACC
IL-6	(F) ACA GCC ACT CAC CTC TTC AGA AC(R) TTT TCT GCC AGT GCC TCT TTG C
IL-1β	(F) TGT ACC TGT CCT GCG TGT TGA AAG(R) CTG GGC AGA CTC AAA TTC CAG CTT
IL-4	(F) TCA TTT TCC CTC GGT TTC AG(R) AGA ACA GAG GGG GAA GCA GT
IL-10	(F) TCA GGG TGG CGA CTC TAT(R) TGG GCT TCT TCT AAA TCG TTC
IFN-γ	(F) ATA TCT TGG CTT TTC AGC TC(R) CTC CTT TTT CGC TTC CCT GT
GAPDH	(F) GAC CCC TTC ATT GAC CTC AAC TAC(R) ATG ACA AGC TTC CCG TTC TCA G

^a^ TNF-α, tumor necrosis factor-alpha; IL-6, interleukin-6; IL-1β, interleukin-1 beta; IL-4, interleukin-4; IL-10, interleukin-10; IFN-γ, interferon-gamma; and GAPDH, glyceraldehyde 3-phosphate dehydrogenase.

**Table 2:** Antibacterial activity of *Lpb. plantarum* Wikim0112, WCFS1, and KACC11451 against foodborne bacterial pathogens.

Pathogens	Inhibition Zone (mm) ± SD ^a^		
	WiKim0112	WCFS1	KACC11451
*E. coli* O157:H7 (ATCC 35150)	18.0 ± 0.5	19.0 ± 0.5	16.0 ± 0.1
*Listeria monocytogenes* (ATCC 15313)	20.0 ± 0.4	18.0 ± 0.2	15.0 ± 0.5
*Salmonella choleraesuis* (KCCM40763)	20.0 ± 0.3	20.0 ± 0.1	16.0 ± 0.2
*Shigella boydii* (KCCM41649)	23.0 ± 0.4	24.0 ± 0.2	21.0 ± 0.2
*Staphylococcus aureus* (ATCC25923)	23.0 ± 0.3	23.0 ± 0.1	20.0 ± 0.3
*Yersinia enterocolitica* (KCCM41657)	23.0 ± 0.2	24.0 ± 0.5	22.0 ± 0.5

^a^ Standard deviation.

**Table 3:** Antibiotic susceptibility of *Lpb. plantarum* Wikim0112, WCFS1, and KACC11451.

Antibiotics	Wikim0112		WCFS1		KACC11451	
	Zone Diameter (mm) ± SD ^a^	Sensitivity	Zone Diameter (mm) ± SD	Sensitivity	Zone Diameter (mm) ± SD	Sensitivity
Ampicillin (AMP)	29.0 ± 1.4	S	39.3 ± 1.3	S	29.7 ± 1.3	S
Chloramphenicol (C)	31.0 ± 0.8	S	30.7 ± 0.9	S	30.7 ± 0.9	S
Clindamycin (CD)	26.3 ± 1.3	S	31.7 ± 1.3	S	12.0 ± 1.6	R
Ciprofloxacin (CIP)	14.3 ± 0.9	I	13.0 ± 1.4	R	13.7 ± 0.5	R
Gentamicin (CN)	18.7 ± 1.3	I	28.7 ± 0.9	S	28.3 ± 1.3	S
Doxycycline (DXT)	28.7 ± 0.9	S	16.3 ± 1.3	I	18.7 ± 0.9	I
Erythromycin (E)	37.0 ± 0.8	S	38.0 ± 1.6	S	33.7 ± 1.3	S
Kanamycin (K)	12.3 ± 1.3	S	31.3 ± 0.5	S	28.3 ± 0.5	S
Penicillin G (P)	29.3 ± 0.9	S	31.3 ± 1.3	S	24.7 ± 0.5	S
Streptomycin (S)	13.3 ± 1.3	R	25.3 ± 1.3	S	23.0 ± 1.4	S
Trimethoprim-sulfamethoxazole (SXT)	29.3 ± 1.6	S	26.7 ± 0.5	S	29.7 ± 1.4	S
Tetracycline (TE)	18.7 ± 1.3	I	12.0 ± 1.3	R	-	R
Vancomycin (VA)	- ^b^	R	-	R	-	R

^a^ Standard deviation; ^b^ Indicates no inhibition zone. The inhibition zones were evaluated according to the Clinical and Laboratory Standards Institute (CLSI) 2012 standards and presented as follows: Susceptible (S) ≥ 20 mm; Intermediate (I) 15–19 mm; Resistant (R) ≤ 14 mm.
